# Predictors of infectious meningitis or encephalitis: the yield of cerebrospinal fluid in a cross-sectional study

**DOI:** 10.1186/s12879-020-05022-6

**Published:** 2020-04-23

**Authors:** Tolga Dittrich, Stephan Marsch, Adrian Egli, Stephan Rüegg, Gian Marco De Marchis, Sarah Tschudin-Sutter, Raoul Sutter

**Affiliations:** 1 Clinic for Intensive Care Medicine, University Hospital Basel, Basel, Switzerland; 2 Medical faculty of the University of Basel, Basel, Switzerland; 3 Division of Clinical Microbiology, University Hospital Basel, Basel, Switzerland; 4 Applied Microbiology Research, Department of Biomedicine, University of Basel, Basel, Switzerland; 5 Clinic for Intensive Care Medicine and Department of Neurology, University Hospital Basel, Basel, Switzerland; 6 Division of Infection Diseases and Hospital Epidemiology, University Hospital Basel, Basel, Switzerland

**Keywords:** Meningitis, Encephalitis, Meningoencephalitis, Cerebrospinal fluid, Neurocritical care

## Abstract

**Background:**

Cerebrospinal fluid (CSF) analyses are recommended in patients with meningitis and/or encephalitis, but evidence regarding its diagnostic yield is low. We aimed to determine predictors of infectious pathogens in the CSF of adult patients presenting with meningitis, and/or encephalitis.

**Methods:**

Consecutive patients with meningitis and/or encephalitis form 2011–17 at a Swiss academic medical care center were included in this cross-sectional study. Clinical, neuroradiologic, and laboratory data were collected as exposure variables. Infectious meningitis and/or encephalitis were defined as the composite outcome.

For diagnosis of bacterial meningitis the recommendations of the European Society of Clinical Microbiology and Infectious Diseases were followed. Viral meningitis was diagnosed by detection of viral ribonucleic or deoxyribonucleic acid in the CSF. Infectious encephalitis was defined according to the International Encephalitis Consortium (IEC). Meningoencephalitis was diagnosed if the criteria for meningitis and encephalitis were fulfilled. Multinomial logistic regression was performed to identify predictors of the composite outcome. To quantify discriminative power, the c statistic analogous the area under the receiver-operating curve (AUROC) was calculated. An AUROC between 0.7–0.8 was defined as “good”, 08–0.9 as “excellent”, and > 0.9 as “outstanding”. Calibration was defined as “good” if the goodness of fit tests revealed insignificant *p*-values.

**Results:**

Among 372 patients, infections were diagnosed in 42.7% presenting as meningitis (51%), encephalitis (32%), and meningoencephalitis (17%). Most frequent infectious pathogens were *Streptococcus pneumoniae*, *Varicella zoster, and Herpes simplex 1&2.* While in multivariable analysis lactate concentrations and decreased glucose ratios were the only independent predictors of bacterial infection (AUROCs 0.780, 0.870, and 0.834 respectively), increased CSF mononuclear cells were the only predictors of viral infections (AUROC 0.669). All predictors revealed good calibration.

**Conclusions:**

Prior to microbiologic workup, CSF data may guide clinicians when infection is suspected while other laboratory and neuroradiologic characteristics seem less useful. While increased CSF lactate and decreased glucose ratio are is the most reliable predictors of bacterial infections in patients with meningitis and/or encephalitis, only mononuclear cell counts predicted viral infections.

**Trial registration:**

ClinicalTrials.gov identifier NCT03856528. Registered on February 26th 2019.

## Background

The high morbidity and mortality of infectious meningitis, encephalitis, and meningoencephalitis and the low specificity of their clinical signs and symptoms have led to a number of studies aiming to generate prediction models for the presence of infectious meningitis and/or encephalitis as summarized by the European Society of Clinical Microbiology and Infectious Diseases (ESCMID) [1] and the Consensus Statement of the International Encephalitis Consortium (IEC) [2]. Despite these efforts, the ESCMID, the European Study Group for Infections of the Brain ESGIB [1], and the Consensus Statement of the IEC [2] concluded that none of the published diagnostic algorithms were reliable to identify patients with infectious meningitis and/or encephalitis in adult patients upon validation in independent cohorts. Most importantly, they maintain that the presence of these infections cannot be proven without examination of the cerebrospinal fluid (CSF). Although microbiologic and CSF analyses are strongly recommended in patients with suspected infectious meningitis and/or encephalitis by international guidelines including the Neurocritical Care Society (NCS) [1–4], evidence from large cohort studies in adults regarding the diagnostic yield of CSF analysis is limited to a small number of studies with various study designs, cohort definitions, and study quality [5–9]. In addition, most studies are restricted to small sample sizes and to the era in which non-culture based diagnostic tests, such as serology and polymerase chain reaction (PCR) were not readily available [10–13]. Moreover, these studies are directed towards the diagnosis of bacterial and/or viral meningitis which is often associated with more impressive and seminal clinical signs and symptoms than infectious encephalitis. As there is a lack of comparison of the predictive strength of CSF analysis and other clinical or neuroradiologic characteristics in this context, clinicians are challenged the regarding early diagnosis, rapid treatment escalation and de-escalation including potentially harmful antimicrobial drugs during the first hours in patients with suspected infectious meningitis and/or meningoencephalitis. Different multiplex PCR assaies and hole genome sequencing approaches for rapid detection of several bacterial and viral infections, promise a paradigm shift in the diagnosis of and may aid in the rapid identification of infectious meningitis and/or encephalitis. However, large clinical studies in the context of meningitis and/or encephalitis are scarce and many hospitals have not implemented this assay yet, or do not provide a 24/7 service, and the turn-around-time is still up to two hours. Hence, there is an urgent need for comprehensive analyses regarding the reliability of independent predictors such as discrimination and calibration in a large number of clinical, neuroradiologic, and microbiologic, as well as non-microbiologic laboratory parameters.

We address this issue by determining independent predictors of the detection of infectious pathogens in adult patients presenting with meningitis, encephalitis, or meningoencephalitgis.

## Methods

### Study design, setting, and ethical approval

This retrospective observational study was performed at a Swiss tertiary academic medical care center (University Hospital of Basel). A care center treating up to 50′000 emergencies per year. To assure the high quality and standardization for the reporting of this observational study, we followed the STROBE (Strengthening the Reporting of Observational Studies in Epidemiology)-guidelines [14]. As the retrospective data collection did not interfere with routine clinical practice and data were safely encoded, the local ethics committee (Ethikkommission Norwest- und Zentralschweiz; No. 2016–01244) approved the study and patients’ consent was waived. The data were anonymized before their use. As all researchers involved in the data collection and analyses were medical employees of the hospital, no additional administrative permissions and/or licenses were acquired by our team to access the data used in our research and no further permission from the hospital was required to access the medical records of patients. We registered the study in the ClinicalTrials.gov registry (ID NCT03856528) on February 26th 2019.

### Selection of participants and collection of data

From January 1st, 2011 to December 31st, 2017, data from clinical, radiological, and laboratory diagnostics were collected consecutively from all patients of at least 18 years of age with the clinical diagnosis of meningitis, encephalitis, or meningoencephalitis. The criteria for the diagnosis of meningitis, encephalitis and meningoencephalitis are presented elsewhere [15]. In short, the diagnosis was based on the patients’ histories and the presence of some or all of the following clinical signs and symptoms: headache, neck stiffness, fever, phono- and/or photophobia. We excluded all patients who did not receive a lumbar puncture for diagnostic purposes. Data were assessed and manually extracted by screening the digital institutional laboratory, microbiologic, and medical history databases. Data were collected and encoded using a predefined digital case report form in Microsoft_®_ Excel 16.1 (Microsoft Corp., Redmond, Washington, USA).

In addition, we collected demographics, such as age, and sex, referral from other hospitals, admission via emergency room, and the Charlson Comorbidity Index to quantify the burden of patients’ comorbidities [16]. We further assessed the suspected initial diagnosis (meningitis and/or encephalitis), the Glasgow coma score on day of diagnosis, and the history of prior disorders of the central nervous system. Time from admission to neuroimaging was defined as the performance of computed tomography and/or magnetic resonance imaging and lumbar puncture. Neuroimaging reports were independently interpreted by two board certified neuroradiologists and screened for the presence and type of brain lesions including signs of cerebral inflammation and intracerebral edema intracerebral, brain abscess, subarachnoid, subdural, and epidural hemorrhage, ischemic stroke, and intracranial tumors. In addition, acute-phase proteins (i.e., albumin and C-reactive protein), lactate, and blood cell counts were assessed on the day of diagnosis of infection. Cerebrospinal fluid was analyzed regarding the counts of white blood cells, and levels of lactate, protein, glucose and the glucose ratio of CSF/serum.

We further assessed the administration of empiric and targeted antimicrobials, the duration of ICU and in-hospital treatment, including intubation, the administration of antiseizure drugs and vasopressors, and the emergence of complications during hospital stay, such as infections, arterial hypotension, epileptic seizures, status epilepticus as defined by the International League Against Epilepsy (ILAE) [17], and organ failure.

Furthermore, in-hospital death, care withdrawal, and functional outcome in survivors (primarily graded by the Glasgow outcome Scale) was assessed to categorize patients into survivors with and without complete neurological recovery.

### Endpoint definition of infectious meningitis and/or encephalitis

The diagnoses of meningitis, encephalitis and meningoencephalitis were systematically reassessed by the investigators using the preestablished frameworks as previously published [15].

In our institution, the diagnosis of viral meningeal infection was based on the detection of viral ribonucleic acid (RNA) or deoxyribonucleic acid (DNA) with PCR in the CSF [18]. The emergence of clinical symptoms as mentioned above was not mandatory, as often only few symptoms are present and the diagnosis of infectious meningitis and/or meningoencephalitis was finally made by the treating physician’s.

The diagnostic workup for bacterial meningitis strictly adhered to the guidelines of the European Society of Clinical Microbiology and Infectious Diseases (ESCMID) [1]. The diagnosis of infectious meningitis was established with the microscopic detection of Gram stained infectious pathogens in the CSF, the detection of aerobic and anaerobic bacterial cultures for 6 days, and/or PCR. The diagnosis of *Borrelia burgdorferi* meningitis was established according to the national guidelines by the detection of intrathecal antibodies [19].

Infectious encephalitis was diagnosed with the detection of a pathogen as described for meningitis in conjunction with the presence of clinical signs of acute encephalopathy as recommended by the International Encephalitis Consortium (IEC) [2]. Acute encephalopathy was defined by lethargy, altered consciousness for at least 24 h, and personality change not sufficiently explained by ischemic, metabolic, and/or other noninfectious cerebral lesions, and more than one of the following: emergence of fever, new neurologic deficits, seizures not previously described, and electroencephalographically or neuroradiologically detected changes not explained by alternative causes.

With the exception of tick-borne encephalitis (“Frühsommer” Meningoencephalitis, FSME) which was diagnosed with positive serology [20], the diagnosis of meningoencephalitis was established in patients presenting signs and symptoms compatible with meningitis and encephalitis.

### Outcomes

Independent predictors of meningitis, encephalitis, and meningoencephalitis with identified infectious pathogens were selected as primary composite outcome. Respective definitions are outlines above.

### Statistical analyses

Missing data was addressed by excluding all data of participants with missing values. As symptoms such as fever, headache, and neck stiffness were inconsistently recorded in the medical records, we decided a priori not to include these variables in all our analyses, thus missing data regarding these variables was not considered an exclusion criteria for this study. Patients were categorized as having or not having identified infectious pathogens as mentioned above. Categorical clinical, laboratory, and radiologic characteristics of these groups were univariably compared using the Chi-square test or the Fisher’s exact test. For the comparison of continuous variables the Shapiro-Wilk test was used to distinguish between normal and abnormal distributions. Variables with normal distributions were analyzed by the Student’s *t* test, non-normally distributed by the Mann-Whitney *U* test. For multiple comparisons (*n* = 30 including all comparisons of Table 1 and the comparisons regarding blood cell counts and chemistry, neuroimaging, and CSF data), the Bonferroni approach was used to adjust for level for significance. The statistical analyses were designed to identify independent predictive factors with sufficient discriminatory power deriving from well-calibrated and cross-validated regression models. Uni- and multivariable multinomial logistic regression analysis were performed to identify variables with an independent association by including all variables differing significantly between the two groups and independent of variables identified as possible confounders in the univariable comparisons.

**Table 1:** Demographics, initial principal diagnosis, infectious pathogens, and comorbidities of patients with meningitis and/or encephalitis with and without identified infectious pathogens

Demographics and admission characteristics	Total cohort (***n*** = 372)		Patients with identified pathogens (***n*** = 159)		Patients without identified pathogens (***n*** = 213)		***p***-value
Age (years; median, IQR)	54	36–71	58	38–72	51	35–70	0.217
Male (n, %)	215	57.8	85	53.5	130	61.0	0.143
Referral from other hospital (n, %)	101	27.2	39	24.5	62	29.1	0.326
Admission via emergency room (n, %)	324	87.1	139	87.4	185	86.8	0.872
Suspected meningitis and/or encephalitis as reason of admission (n, %)	262	70.4	113	71.1	149	69.9	0.815
Glasgow coma score on day of diagnosis (median, IQR)	14	7–15	13	4–15	14	11–15	0.004
**Initial clinical diagnosis**							
Meningitis (n, %)	170	45.7	81	50.9	89	41.8	0.193
Encephalitis (n, %)	136	36.6	51	32.1	85	39.9	
Meningoencephalitis (n, %)	66	17.7	27	17.0	39	18.3	
**Infectious pathogens**							
Bacterial (n, %)	71	19.1	71	44.6			
*Streptococcus pneumoniae* (n, %)	27	7.3	27	17.0			
*Borrelia burgdorferi* (n, %)	14	3.8	14	8.8			
*Neisseria meningitidis* (n, %)	5	1.3	5	3.1			
Others (n, %) (*Staphylococcus aureus, Streptococcus pyogenes, Streptococcus agalactiae, Haemophilus influenzae, Mycobacterium tuberculosis, Enterobacter cloacae, Enterococcus faecalis, Listeria monocytogenes)*	25	6.7	25	15.7			
Viral (n, %)	85	22.8	85	53.5			
*Varicella zoster* (n, %)	25	6.7	25	15.7			
*Herpes simplex 1 & 2* (n, %)	22	5.9	22	13.8			
*Enterovirus* (n, %)	21	5.6	21	13.2			
Others (n, %) (*FSME-virus, Human herpesvirus 6, JC-virus)*	17	4.6	17	10.7			
Protozoal (all *Toxoplasma gondii*; n, %)	3	0.8	3	1.9			
**Comorbidities**							
Charlson Comorbidity Index [16] (median, IQR)	0	0–2	0	0–2	0	0–2	0.913
Immunosuppression (n, %)	111	29.8	58	36.5	53	24.9	0.017
History of drug abuse (n, %)	27	7.3	11	6.9	16	7.5	0.827
History of prior CNS disorders (n, %)	149	40.1	67	42.1	82	38.5	0.478
Epileptic disorder (n, %)	65	17.5	29	18.2	36	16.9	0.737
Prior meningitis and/or encephalitis (n, %)	28	7.5	15	9.4	13	6.1	0.228
Prior autoimmune CNS disease (n, %)	3	0.8	2	1.3	1	0.5	
Prior Dementia (n, %)	11	3.0	5	3.1	6	2.8	
Prior leukoencephalopathy (n, %)	46	12.4	22	13.8	24	11.3	0.457
Prior stroke (n, %)	23	6.2	4	2.5	19	8.9	0.015
Prior intracranial hemorrhage (n, %)	13	3.5	7	4.4	6	2.8	0.410
Prior neurodegenerative disorder other than dementia (n, %)	7	1.9	2	1.3	5	2.4	
Prior brain tumor (n, %)	8	2.2	7	4.4	1	0.5	0.023

*IQR* interquartile range, *CNS* central nervous system, *CSF* cerebrospinal fluid, *FSME* “Frühsommer”-Meningoenzephalitis; **Bold*p*-values indicate significance set at a*p*-value of < 0.002 after correction for multiple comparisons (Bonferroni)**

All continuous variables were analyzed using the Mann-Whitney *U* test

To identify variables independently (i.e., controlling for potential confounders) associated with the presence of infectious meningitis and/or encephalitis, stepwise logistic regression with forward and backward selection (with elimination at an α-level of < 0.05) were applied. To select variables that were most predictive, we further performed lasso (least absolute shrinkage and selection operator) regression, a shrinkage method, shrinking coefficient estimates of predictors with little or no predictive value to zero (an odds ratio of 1) [21].

To quantify discriminative power, the c statistic analogous the area under the receiver-operating curve (AUROC) was calculated. An AUROC between 0.7–0.8 as “good”, 08–0.9 as “excellent”, and > 0.9 as “outstanding” as defined elsewhere [22]. Hosmer-Lemeshow and Pearson’s *χ*^2^ goodness-of-fit tests were applied to check the multivariable logistic regression models and assess calibration. Calibration was defined as “good” if the goodness of fit tests revealed insignificant *p*-values.

All analyses were performed with the statistical software STATA® 15.1 (Stata Corp., College Station, TX, USA).

## Results

**Fig. 1:**
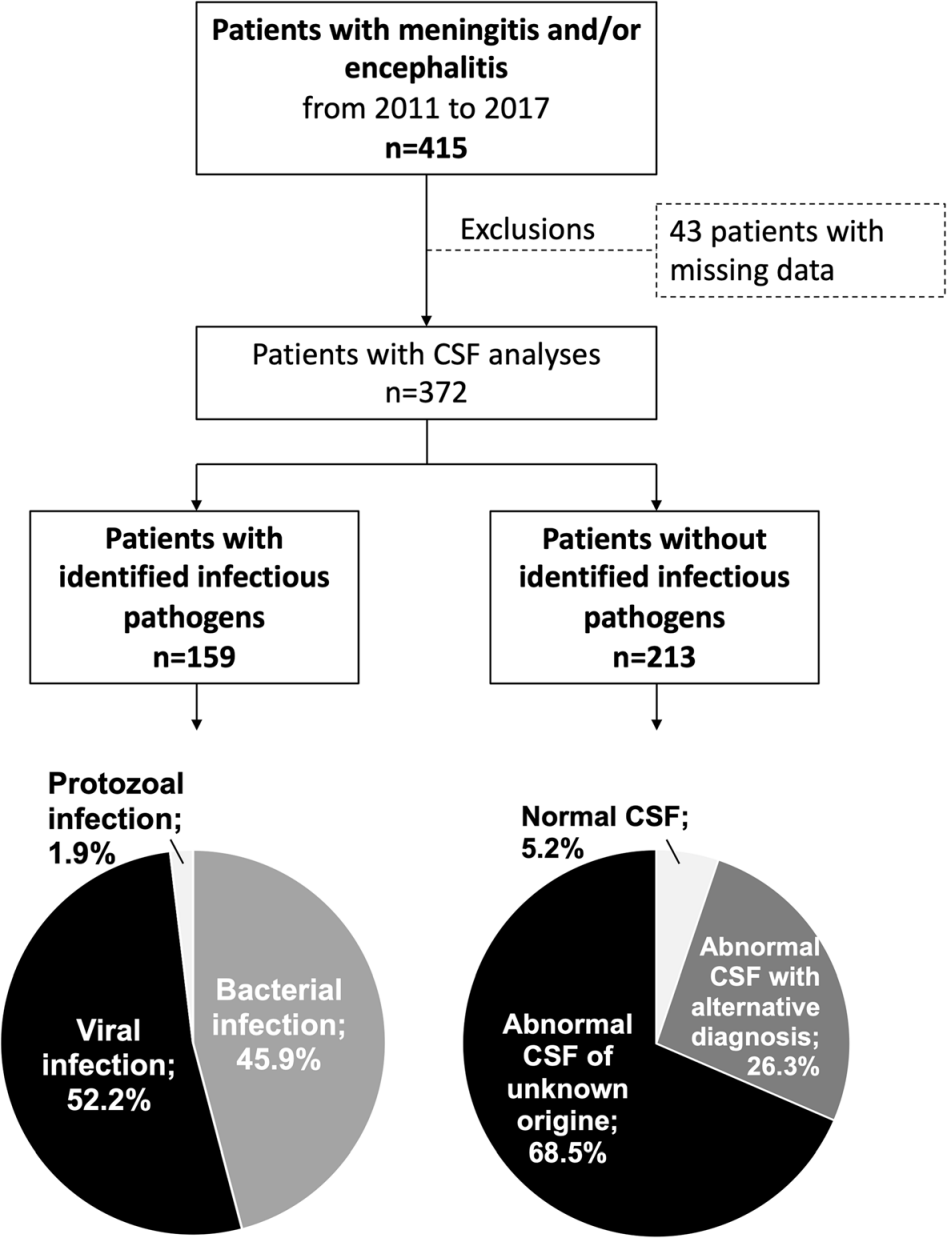
Flow chart. CSF = cerebrospinal fluid. 26.3% (*n* = 56) alternative CNS pathologies: 10 autoimmune/paraneoplastic encephalitides; 7 strokes; 3 intracranial hemorrhages; 3 traumatic brain injuries; 3 septic encephalopathies; 3 brain tumors; 3 prion diseases; 11 others

Out of all 415 consecutive adult patients with meningitis and/or encephalitis, data were missing in 43 were excluded from further analyses (Fig. 1). The remaining 372 patients had a median age of 54 years (interquartile range [IQR] 36–70) and a pathogen was identified in 42.7%. Alternative diagnoses in patients in whom no pathogen could be identified are compiled in Fig. 1. Demographics, initial principal diagnosis, infectious pathogens, and comorbidities are presented in Table 1. The initial clinical presentation was most frequently interpreted as meningitis, followed by encephalitis, and meningoencephalitis. Median time from admission to neuroimaging was 2.8 h (IQR 1.1–17.3) in patients with and 3.4 h (IQR1.8–8.1) in patients without diagnosed infections. Median time from imaging to lumbar puncture was 2 h (IQR 1.1–6.5) in patients with infections and 3 h (IQR 1.4–14.4) in patients without. Most frequent infectious pathogens were *Streptococcus pneumoniae*, *Varicella zoster, and Herpes simplex 1&2* (Table 1)*.* Table 2 presents the comparisons of blood cell counts and chemistry on day of diagnosis, neuroimaging, cerebrospinal fluid data, treatment characteristics, complications, and outcomes of patients with meningitis and/or encephalitis with and without identified infectious pathogens. Empiric antimicrobial treatment was started in 86.8% of all patients and in 95% of patients with identified infectious pathogens. 8/159 patients with identified pathogens (2 patients with bacterial and 6 with viral infections) did not receive empiric, but subsequent targeted antimicrobial medication. All received targeted but no empiric antimicrobial treatment. Median time from admission to empiric antimicrobial treatment was 3.7 h (IQR 1.4–7.8). After correction for multiple comparisons, the only predictors of infections were CSF data available before microbiologic workup. Multivariable logistic regression models for the prediction of infectious meningitis and/or encephalitis by CSF data are presented in Table 3**.** While increased lactate concentrations, and decreased glucose ratio (CSF/serum) in the CSF were independently predictive for bacterial infection, elevated CSF mononuclear cells were the only consistent predictors of viral infections.

**Table 2:** Blood cell counts and chemistry on day of diagnosis, neuroimaging, cerebrospinal fluid data, treatment characteristics, complications, and outcomes of patients with meningitis and/or encephalitis with and without identified infectious pathogens

	Total cohort (***n*** = 372)		Patients with identified pathogens (***n*** = 159)		Patients without identified pathogens (***n*** = 213)		***p***-value
**Blood cell counts and chemistry on day of diagnosis**							
White blood cell count (× 10^9^/l; median, IQR)	8.4	6.6–12.0	9.1	7.0–13.7	8.2	6.7–11.6	0.060
C-reactive protein (mg/l; median, IQR)	7.8	1.7–48.3	16.8	3.5–97.5	5.9	1.3–34.2	0.002
Albumin (g/l; median, IQR)	34	30–38	33	29–38	34	30–38	0.245
Lactate (mmol/l; median, IQR)	1.2	0.9–1.7	1.3	0.9–1.9	1.2	0.9–1.6	0.136
**Neuroimaging**							
Neuroimaging performed (n, %)	335	90.1	140	88.1	195	91.6	0.265
Computed tomography performed (n, %)	247	66.4	112	70.4	135	63.4	0.154
Magnetic resonance imaging performed (n, %)	237	63.7	84	52.8	153	71.8	**< 0.001**
Brain lesions on neuroimaging in patients with imaging (n, % patients with imaging)	203	4.6	92	65.7	111	56.9	0.104
Brain edema in patients with imaging (n, % patients with imaging)	39	10.5	19	13.6	20	10.3	0.351
Brain inflammation in patients with imaging (n, % patients with imaging)	68	18.3	35	25.0	33	16.9	0.070
**Cerebrospinal fluid data**							
White blood cells (× 10^6^/l; median, IQR)	65	10–261	171	43–575	33	5–122	**< 0.001**
Polynuclear cells (×10^6^/l; median, IQR)	5	0.3–37.4	16	3–187	1	0–12	
Mononuclear cells (×10^6^/l; median, IQR)	39	6–139	81	16–217	21	4–90	
Protein (mg/l; median, IQR)	807	497–1449	1650	629–2470	684	449–1111	**< 0.001**
Lactate (mmol/l; median, IQR)	2.4	1.8–3.5	3	2.1–7.5	2.1	1.6–2.8	**< 0.001**
Glucose (mmol/l; median, IQR)	3.2	2.6–3.8	2.9	1.9–3.5	3.3	2.9–3.9	**< 0.001**
Glucose ratio (CSF/serum; median, IQR)	0.6	0.5–0.6	0.5	0.4–0.6	0.6	0.5–0.7	**< 0.001**
**Treatment**							
Hospital stay (days; median, IQR)	11	5–19	14	6–21	9	4–17	0.015
Treatment on ICUs (n, %)	141	37.9	69	43.4	72	33.8	0.059
ICU stay of patients in ICUs (days; median, IQR)	3	2–7	4	2–7	3	2–7	0.208
Mechanical ventilation (n, %)	43	11.6	29	18.2	14	6.6	**< 0.001**
Empiric antimicrobial treatment (n, %)	323	86.8	151	95.0	172	80.8	**< 0.001**
Antiseizure drugs (n, %)	121	32.5	51	32.1	70	32.9	0.872
Vasopressors (n, %)	4411.8		28	17.6	16	7.5	0.003
**Complications**							
Arterial hypotension (n, %)	44	11.8	28	17.6	16	7.5	0.003
Epileptic seizures (n, %)	25	6.7	14	8.8	11	5.2	0.165
Status epilepticus (n, %)	20	5.4	7	4.4	13	6.1	0.643
Aspiration pneumonia (n, %)	13	3.5	6	3.8	7	3.3	0.785
Brain herniation (n, %)	6	1.6	4	2.5	2	0.9	0.408
**In-hospital outcomes**							
Death (n, %)	11	3.0	3	1.9	8	3.8	0.365
Care withdrawal (n, %)	7	1.9	3	1.9	4	1.9	1.000
Return to premorbid baseline (n, %)	236	63.4	102	64.2	134	62.9	0.806

*IQR* interquartile range, *ICU* intensive care unit; **Bold*p*-values indicate significance set at a*p*-value of < 0.002 after correction for multiple comparisons (Bonferroni)**

All continuous variables were analyzed using the Mann-Whitney *U* test

**Table 3:** Uni- and multivariable logistic regression models for the prediction meningitis and/or encephalitis with identified infectious pathogens by CSF parameters (excluding protozoal infections)

**Logistic regression**										
	Univariable			Multivariable			Hosmer-Lemeshow		Pearson’s	
	OR	95% CI	***p***-value	OR	95% CI	***p***-value	***X***^**2**^	***p***-value	***X***^**2**^	***p***-value
**CSF parameters for infection**										
White blood cells (per10^6^/l)	1.00	0.99–1.00	0.283	1.00	0.99–1.00	0.141	13.50	0.096	305.7	0.321
Protein (per mg/l)	1.01	1.00–1.01	**< 0.001**	0.99	0.99–1.00	0.852				
Lactate (per 0.1 mmol/l)	1.29	1.18–1.42	**< 0.001**	1.11	0.93–1.34	0.254				
Glucose ratio (per 0.1unit)	0.01	0.01–0.06	**< 0.001**	0.05	0.01–0.37	**0.003**				
**Multinomial logistic regression**										
	Univariable			Multivariable						
	RR	95% CI	***p***-value	RR	95% CI	***p***-value				
**CSF parameters for bacterial infection**										
White blood cells (per10^6^/l)	1.00	1.00–1.01	**0.039**	0.58	0.18–1.89	0.368				
Polynuclear cells (per 10^6^/l)	1.01	1.00–1.01	**< 0.001**	1.72	0.53–5.60	0.368				
Mononuclear cells (per 10^6^/l)	1.01	1.00–1.01	**< 0.001**	1.72	0.53–5.61	0.367				
Protein (per mg/l)	1.01	1.00–1.01	**< 0.001**	1.00	0.99–1.00	0.982				
Lactate (per 0.1 mmol/l)	1.55	1.38–1.75	**< 0.001**	1.34	1.04–1.71	**0.021**				
Glucose ratio (per 0.1unit)	0.01	0.00–0.01	**< 0.001**	0.01	0.00–0.17	**0.003**				
**CSF parameters for viral infection**										
White blood cell count (per10^6^/l)	1.00	0.99–1.00	0.103	0.62	0.30–1.29	0.203				
Polynuclear cells (per 10^6^/l)	0.99	0.99–1.00	0.208	1.61	0.77–3.33	0.204				
Mononuclear cells (per 10^6^/l)	1.01	1.00–1.01	**< 0.001**	1.62	0.78–3.36	0.198				
Protein (per mg/l)	1.00	0.99–1.00	0.634	1.00	1.00–1.00	0.925				
Lactate (per 0.1 mmol/l)	1.02	0.87–1.18	0.830	1.00	0.76–1.30	0.997				
Glucose ratio (per 0.1unit)	0.09	0.01–0.60	**0.012**	0.10	0.10–1.02	0.052				
**Multinomial logistic regression**										
	Forward selection			Backward selection			Hosmer-Lemeshow		Pearson’s	
	RR	95% CI	***p***-value	RR	95% CI	***p***-value	***X***^**2**^	***p***-value	***X***^**2**^	***p***-value
**CSF parameters for bacterial infection**										
White blood cells (per10^6^/l)		NS			NS					
Polynuclear cells (per 10^6^/l)		NS			NS					
Mononuclear cells (per 10^6^/l)		NS			NS					
Protein (per mg/l)		NS			NS					
Lactate (per 0.1 mmol/l)	1.35	1.12–1.64	**0.002**	1.35	1.12–1.64	**0.001**	6.58	0.583	135.8	0.14
Glucose ratio (per 0.1unit)	0.01	0.00–0.23	**0.004**	0.10	0.00–0.23	**0.004**				
**CSF parameters for viral infection**										
White blood cell count (per10^6^/l)		NS			NS					
Polynuclear cells (per 10^6^/l)		NS			NS					
Mononuclear cells (per 10^6^/l)	1.01	1.00–1.01	**< 0.001**	1.01	1.00–1.01	**< 0.001**	9.46	0.305	194.1	0.39
Protein (per mg/l)		NS			NS					
Lactate (per 0.1 mmol/l)		NS			NS					
Glucose ratio (per 0.1unit)		NS			NS					

*CSF* cerebrospinal fluid, *OR* odds ratio, *CI* confidence interval, *NS* not selected

**Bold*p*-values indicate significance set at < 0.05**

### Discrimination and calibration of non-microbiologic CSF data

**Fig. 2:**
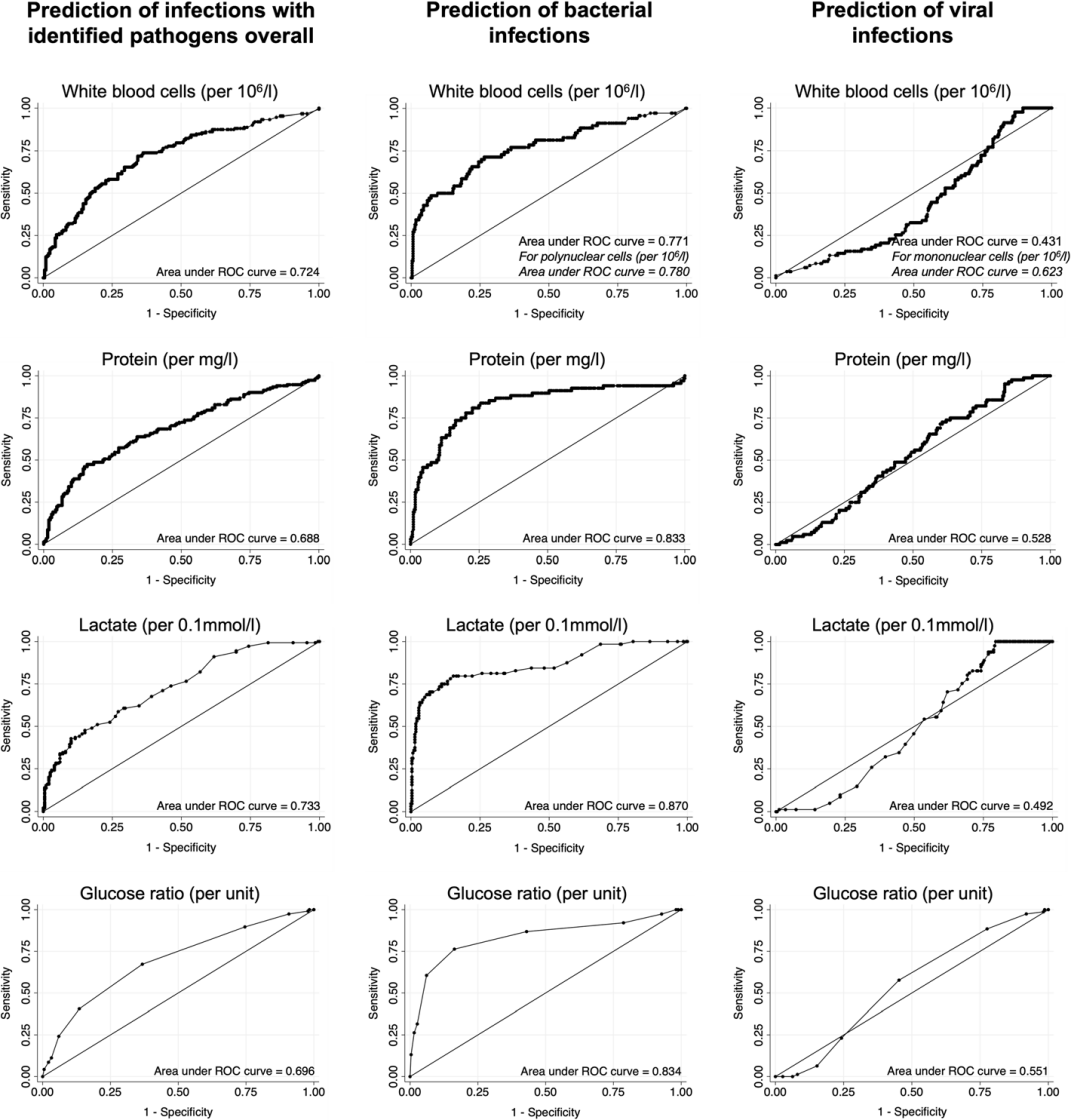
Receiver operating characteristic analyses for the prediction of meningitis and/or encephalitis with identified infectious pathogens by CSF parameters (excluding protozoal infections). CSF = cerebrospinal fluid

Figure 2 presents the receiver operating characteristic analyses for the prediction of infectious meningitis and/or encephalitis by CSF parameters. While the AUROCs of these CSF predictors of infection (regardless of the infectious pathogens) were between 0.688 and 0.733, the AUROCs for the prediction of a bacterial infection were higher between 0.780 and 0.870. The lowest AUROCs were seen for the prediction of viral infections with the exception of an AUROCs of 0.780 of increased mononuclear cells.

For the presence of infections, decreasing glucose ratio was the only independent predictors with an AUROC 0.696 (95% CI 0.64–0.75). The AUROC of the two independent predictors of bacterial infections were 0.870 (95% CI 0.81–0.92) for elevated lactate concentrations, and 0.834 (95% CI 0.75–0.92) for decreased glucose ratio. Increased CSF lactate levels and decreased glucose ratio were well calibrated predictors as expressed by a non-significant goodness-of-fit test (Table 3). For viral infections, the only consistent predictors were elevated mononuclear cells with an AUROC of 0.623 (95% CI 0.55–0.69) and good calibration (Table 3).

## Discussion

Our study identified decreased glucose ratio, as the only independent predictors of infections in adult patients with meningitis and/or encephalitis and further provides quality metrics such as values for discrimination and calibration among a large number of well-defined clinical, laboratory, and neuroradiologic characteristics. Elevated CSF lactate concentrations and decreased glucose ratio were the only independent and discriminative predictors of bacterial infections in patients with meningitis and/or encephalitis, while elevated mononuclear cells were the only predictors for viral infections. At first glance, the odds ratio of 1.34 for the detection of bacteria by CSF lactate concentration seems small. However, it has to be taken into account that they are given for each increase in mmol/l.

While our results may be considered as common clinical knowledge, data adding to the surprisingly limited body of evidence in this context is scarce and urgently needed.

Demographics and clinical characteristics in our cohort are similar to those reported in several other studies regarding infectious meningitis and/or encephalitis including age [7, 9, 11], level of consciousness [7, 8], proportion of immunosuppressed patients [6, 7], identified infectious pathogens [7, 8, 11], epileptic complications [7, 8], time to neuroimaging and lumbar puncture [8], empiric antimicrobial treatment [8, 23], length of hospital stay [11], and outcome [8, 23] – indicating that our cohort is comparable to other populations of adults in this context. The proportion of patients infected by the most frequently identified infectious pathogens are also in line with several earlier studies, mainly reporting *Streptococcus pneumoniae, Varicella zoster, and Herpes simplex 1&2* as most frequent infectious pathogens [7, 8, 11]*.* Although time to neuroimaging, lumbar puncture and start of empiric antimicrobial treatment was comparable to other recent studies, it is still unacceptably long, as increasing time from admission to administration of antimicrobial treatment is associated with increased mortality [24] and it seems likely that with delay of diagnostic workup, time to treatment may increase. Interestingly, autoimmune and paraneoplastic encephalitides represented the most frequent alternative non-infectious etiology of meningitis and/or encephalitis.

Uni- and multivariable analyses revealed that CSF data were the only independent and discriminative predictors of the presence of an infection in this setting. These findings add credence to the limited body of evidence demonstrating that data from early CSF analyses prior to microbiologic workup in a cohort of adult patients with meningitis and/or encephalitis provide important diagnostic information. Increased lactate concentrations, and decreased glucose ratio (CSF/serum) in the CSF were identified as independent predictors of bacterial infection with good discrimination. Regarding viral infections increased CSF mononuclear cells were the only consistent predictors, however, without showing good discrimination between presence and absence of these infections. Among all discriminative CSF parameters, the only predictors with both good discrimination and calibration were elevated lactate concentrations and decreased glucose ratio for the prediction of bacterial infection.

This finding is in line with few early studies describing CSF lactate levels being the most discriminative parameter to differentiate between aseptic and bacterial or viral meningitis [7, 10–13]. However, the fact that non-culture based diagnostic tests, such as serology and PCR, were not as readily available, resulting in potentially missed diagnoses of bacterial meningitis. In addition, analyses regarding quality metrics of the reliability of independent predictors such as discrimination and calibration were lacking in all studies. While reliable discrimination represents the ability of a test to diagnose patients with and without a specific disease, precise calibration portends that a calculated score forecasting a probability of an event in a group of patients turns out to be identical to the actual events emerging in that group.

In contrast to the prediction of bacterial infection, both glucose ratio as the independent predictor of infection overall and increased CSF mononuclear cells as predictors of viral infections showed good calibration, but insufficient discrimination.

It is worrisome that the discrimination of CSF data regarding the presence of infection regardless of the type of pathogen (i.e., bacterial or viral) and of isolated viral infection was insufficient and that no other clinical, non-microbiologic laboratory, and neuroradiologic parameters showed independent and discriminative prediction of infections.

## Limitations

The single-center observational design limits the generalizability of this study. However, the demographics and clinical characteristics of our study population are similar to those reported in other studies on infectious meningitis and/or encephalitis indicating that our cohort is representative and comparable to other cohorts of adults with meningitis and/or encephalitis. Unfortunately, analyses regarding clinical symptoms, such as fever, headache, and neck stiffness were not possible because of missing or inconclusive data. However, as it is well known that the reliability of these symptoms regarding the presence of infectious meningitis and/or encephalitis is low, it is unlikely that this shortcoming has affected our results. In addition, the sensitivity and specificity of the microbiological methods to confirm an infection (i.e., identify a pathogen) may have changed over the study period and affected our results. As testing methodologies for detection of viral and bacterial pathogens changed over the study period, potentially influencing our findings. However, as culture methodologies for detection of bacteria were constant over time and the detection of viruses was based on PCR diagnostics over time, I significant bias seems unlikely. Given that the case definitions and expert consensuses have been revised during the study period, there is a risk of information bias. However, our study aimed to investigate the use of clinical, CSF, and neuroradiologic assessments in patients in whom the clinician decided to perform lumbar puncture in the setting of clinically suspected infection of the central nervous system. Hence, as clinicians suspect central nervous system infection in the clinical context and not as a result of decisions of experts (i.e., it seems very likely that a clinician in 2011 has suspected central nervous system infection based on the clinical context the same way as a clinician in 2017), the risk of information bias does not seem relevant.

As all 43 patients did not have a lumbar puncture, a potential selection bias cannot be excluded. However, as 32 (74%) of the 43 excluded patients did not have a lumbar puncture due to contraindications, such as anticoagulation, intracerebral masses, intracerebral edema, and others, further analyses regarding the question whether CSF analyses are generalizable to such patients with contraindications of lumbar puncture are obsolete, as these patients will never receive any CSF analyses in this context. Hence a potential selection bias can be ignored from a clinical standpoint.

## Conclusions

Our study reveals that CSF data available prior to microbiologic workup and confirmation of infection may guide clinicians when infection is suspected while other laboratory and neuroradiologic characteristics seem less useful. While elevated CSF lactate concentration and decreased glucose ratios are the only independent and discriminative predictors of bacterial infections among clinical, radiologic, and laboratory parameters in patients with meningitis and/or encephalitis, only elevated mononuclear cells predicted viral infections. While our results may be considered as common clinical knowledge, data adding to the surprisingly limited body of evidence in this context is scarce and urgently needed. Further external validation of the predictors identified is the step prior to the generation of prediction scores aiming to assist the clinician in daily decision making.

The lack of predictors of infection in adult patients with meningitis and/or encephalitis indicates that clinicians are urged to withhold decision regarding the termination of empiric antimicrobial treatment prior to the microbiologic workup in patients without increased CSF lactate levels.

## Data Availability

The datasets used and/or analyzed during the current study are available from the corresponding author on reasonable request.

## Abbreviations

AUROC: Area Under the Receiver-Operating Curve
CSF: Cerebrospinal Fluid
DNA: Deoxyribonucleic Acid
ESCMID: Clinical Microbiology and Infectious Diseases
ESGIB: European Study Group for Infections of the Brain
FSME: “Frühsommer” Meningoencephalitis
HIV: Human Immunodeficiency Virus
IEC: Consensus Statement of the International Encephalitis Consortium
IQR: Interquartile Range
ILAE: International League Against Epilepsy
NCS: Neurocritical Care Society
PCR: Polymerase Chain Reaction
RNA: Ribonucleic Acid
STROBE: Strengthening The Reporting of Observational Studies in Epidemiology
